# Exploring diet-microbiota interactions and therapeutic nutrition management in inflammatory bowel disease

**DOI:** 10.52601/bpr.2024.240050

**Published:** 2025-06-30

**Authors:** Xinran Wang, Yiran Wang, Lulu Sun

**Affiliations:** 1 State Key Laboratory of Female Fertility Promotion, Department of Endocrinology and Metabolism, Peking University Third Hospital, Beijing 100191, China; 2 Institute of Advanced Clinical Medicine, Peking University, Beijing 100191, China

**Keywords:** Intestinal bowel disease (IBD), Gut microbiota, Microbial metabolites, Exclusive enteral nutrition (EEN)

## Abstract

Intestinal bowel disease (IBD) is a chronic, early-onset, recurrent gastrointestinal immune-related disease that has become globalized. Although the combination of genetic, environmental, and immunological factors leads to intestinal inflammation and barrier damage, the etiology of IBD is not clearly defined. In recent years, diet-microbiota interactions have been widely studied for their potential in pathogenesis and treatment for IBD. Meanwhile, the significant efficacy of exclusive enteral nutrition (EEN) has been observed in clinical practice with modulation in gut microbiota, but the specific mechanisms and optimization measures remain challenging. Therefore, we first describe the development of existing microbial research techniques and the perspectives that can be broadened. We then synthesize findings on how dietary components impact IBD progression and treatment through microbiota. Finally, based on correlating clinical and basic experiments, we summarize the current status and potential mechanisms of EEN for treating IBD, especially the contradictory points encountered in its application.

## INTRODUCTION

Intestinal bowel disease (IBD), which consists of two main subtypes, Crohn's disease (CD) and ulcerative colitis (UC), is a heterogeneous, chronic, relapsing, early-age-of-onset, idiopathic immunologic disease of the gastrointestinal tract (Dolinger *et al.* 2024; Le Berre *et al.* 2023). From the emergence of compound prevalence in the West to an incremental incidence in the newly industrialized countries, IBD has become a global disease resulting in tremendous socioeconomic burden and devastation of individual lives (Kaplan 2015; Kaplan and Windsor 2020). Although the etiology remains incompletely understood, genetic susceptibility, environmental triggers, and their interplay, compounded by microbial dysbiosis, and immune dysregulation, are crucial culminating in mucosal barrier dysfunction and persistent inflammation (Ananthakrishnan *et al.* 2017; Kan *et al.* 2024; Noble *et al.* 2023). Specifically, dietary constituents can serve as environmental modulators that shape the intricate interplay between immune system homeostasis and microbial ecology (Witkowski *et al.* 2018). Existing drugs for IBD have limitations in efficacy and side effects, necessitating the discovery of new molecular targets and companion biomarkers to advance more effective and precise therapeutic strategies (Honap *et al.* 2024). Nowadays, diet interventions have garnered widespread attention and hold value in clinical recommendations (Hashash *et al.* 2024). However, there is a conspicuous gap in exploring mechanisms between clinical and preclinical trials, which could provide a deeper understanding of pathogenesis and therapy. Herein, we review microbiota-related mechanisms of current dietary interventions for IBD. In particular, we highlight the need for conceptual adjustments in preclinical design, addressing the inconsistencies observed in clinical trials from the objective standpoints, together with the broader horizons that technological development ought to give us, which help capture key points contributing to therapy optimization.

## DIETARY EFFECTS ON GUT MICROBIOTA AND IBD

The gut microbiota refers to the vast assemblage of microorganisms inhabiting the intestinal ecosystem. It predominantly consists of bacteria, viruses, fungi, and archaea (Lynch and Pedersen 2016), which, along with metabolites play crucial roles in gut ecology for mucosal immune formation and maturation (Shi *et al.* 2017), nutrient digestion and absorption (Tang *et al.* 2021), maintaining intestinal barrier integrity (Leonardi *et al.* 2022; Paone and Cani 2020), and also act as signaling molecules in the gut-organ axis with implications for global physiology (Lynch and Pedersen 2016). Still, it is also a double-edged sword in specific contexts (Walker and Hoyles 2023).

Diet, as an exogenous origin for microbiota in the gut microenvironment, significantly impacts gut microbiota homeostasis, which is essential for preventing and treating IBD (Perler *et al.* 2023). In the following, we list the relevant technologies and findings of diet-induced microbiota changes and analyze the important mediating role of microbiome between diet and host by listing the influence of Western diet components on IBD.

### Advanced tools that identify the “modulators”

Due to the development of high-throughput omics technologies, the important link between dysbiosis, referring to alterations in the composition (metagenome) and function (metabolome) of the microbiome, and the pathogenesis of IBD has been widely demonstrated (Sartor and Wu 2017). Especially, there is an emerging number of reports on gut mycobiome yet with the greater uncharted territory (Table 1).

**Table 1:** Reports on gut mycobiome in IBD

Reference	Techniques	Sample	Model/Cohort	Microbial shifts
Imai *et al.* 2019	16S rRNA + ITS	Stool	Inactive UC (*n* = 18), inactive CD (*n* = 20), and healthy control (*n* = 20)	*Saccharomyces* (in healthy control)↑; *Candida* (in CD)↑; the correlation between bacterial and fungal taxa↑
Jangi *et al.* 2024	ITS2	Stool	IBD (*n* = 421): clinical activity (*n* = 104) and clinical remission (*n* = 317)	*Candida* is significantly associated with clinical activity; *Candida* relative abundance was positively correlated with *Parabacteroides diastonis* (+0.16, *p* < 0.05), *Faecalibacterium prausnitzii* (+0.14, *p* < 0.05), and *Bacteroides dorei*; but this correlation is disrupted in flare
Hsia *et al.* 2023	ITS	Stool	UC (*n* = 98): across endoscopic activity (*n* = 43), endohistologic activity (*n* = 41), and biologic exposure (*n* = 82)	*Saccharomyces* and *Candida*↑
Catalán-Serra *et al.* 2024	ITS1	Stool	UC (*n* = 52), CD (*n* = 37), and healthy control (*n* = 22)	*Ascomycota/Basidiomycota*↓ (especially in flare)
Sokol *et al.* 2017	16S rRNA + ITS2	Stool	IBD (*n* = 235) and healthy control (*n* = 38)	*Basidiomycota/Ascomycota*↑ (especially in flare) ; *Saccharomyces cerevisiae*↓; *Candida albicans*↑
Jain *et al.* 2021	qPCR of ITS	Murine mucosal wounds and patient ileal biopsies	Mice injured by colonic biopsies and treated with antibiotics to impair healing; control mice injured but not treated with antibiotics	*Debaryomyces hansenii* abundance in mucosal wounds of antibiotic-treated mice compared with control subjects↑
Lewis *et al.* 2017	Shotgun whole metagenome sequencing	Stool	Pediatric active CD (*n* = 90), healthy pediatric control subjects (*n* = 26)	The abundance of *Candida utiliz*, *Saccharomyces cerevisiae*, *Clavispora lusitaniae*, *Candida* *albicans*, and *Kluyveromyces marxianus* in active CD, which decreased following eight weeks of exclusive enteral nutrition↑
Limon *et al.* 2019	ITS1	166 samples representing the sigmoid colon and cecum	CD patients and healthy controls undergoing screening colonic endoscopy	*Malassezia restricta*↑
16S rRNA: 16S ribosomal RNA; ITS: internal transcribed spacer; qPCR: quantitative real-time PCR				

Fungal and viral sequences have been found to comprise a mere 0.1% of the fecal microbiome gene catalog (Qin *et al.* 2010). Achieving enhanced resolution in their detection mandates deep sequencing, a procedure that is both indispensable and costly. It is a gratifying development to report that a fungal enrichment protocol followed by shotgun metagenomic sequencing, a frugal and efficient combination, achieved the detection of *Debaryomyces hansenii*, which is associated with the dysregulation of mucosal healing in CD (Xie *et al.* 2023). Parallel to this, the strategic filtration method based on volume difference pre-sequencing offers a dependable approach to discerning viroid particles (Kleiner *et al.* 2015). This magnifying glass-like perspective can compensate for biases that may arise from being overshadowed by bacterial dominance during sequencing. However, biases are omnipresent in sequencing-based research, which can even vary depending on the sampling time and source (Allaband *et al.* 2024; Frau *et al.* 2021), yet these inspire the exploration of mechanisms behind gut pathology and circadian rhythms in the microbiota. In this light establishing a uniform experimental design appears to be unnecessary, what is important is to be frank about the differences and to analyze the underlying causes.

However, updated databases and analytical tools can somewhat reduce the impediments to investigating fungi and viruses. For instance, the CGF catalog constructed by fecal culture and sequencing of healthy people has greatly increased the enteric fungal genome resources, which has been validated in animal experiments after collaborating with other public databases to mine IBD-related microbiome (Yan *et al.* 2024). Similarly, the database-based analytics pipeline has been updated in recent years (Větrovský *et al.* 2020; Xie and Manichanh 2022). Still, these methods utilize essentially culture-based sequencing. Several studies in recent years have successfully expanded viral databases with bulk and/or VLP-enriched metagenomes or single-virus sequencing approaches (Tun *et al.* 2024), demonstrating the contribution that culture-independent sequencing technologies can bring to the exploration of the microbiome. In contrast, specific IBD-related microbial profiles can often be depicted from clinical cohort studies, such as pathobionts (Gilliland *et al.* 2024), clusters with prognostic relevance (Yilmaz *et al.* 2019) and morbidity trends (Raygoza Garay *et al.* 2023). However, summarizing standardized microbial signatures remains challenging (Walker and Hoyles 2023), which not only reflects the dynamic and heterogeneous nature of the disease. Whereas fungi have greater fluctuations in composition and function than bacteria (Xie *et al.* 2023), and viruses are characterized by genome instability and variation (Guzzo *et al.* 2022). Although metagenomics combined with network analysis additionally provides interkingdom information, it can only predict functional potential, while transcriptomics and proteomics can reflect actual pathways and functions, particularly, spatial transcriptomics portrays the microbiome in a three-dimensional perspective, reflecting their community behavior and interactions with the host in the niche (Saarenpää *et al.* 2024; Yang *et al.* 2023; Zhuxia and Guangdun 2022). Metabolomics is profoundly significant due to the metabolic output from the entire gut ecology (Krautkramer *et al.* 2021), such as fecal untargeted metabolomics could investigate the molecular mechanisms of microbial metabolites and IBD pathogenesis (Vich Vila *et al.* 2024).

Recent multi-omics correlation analysis reveals that microbiome and metabolome do not directly correlate (Franzosa *et al.* 2019). The study identified numerous unclassified microbial metabolites and suggested using cluster analysis of guilt by association to characterize them (Franzosa *et al.* 2019). This mindset has practical value, such as focusing on the meta-mass shift in the molecular network and discovering novel bile acid conjugates through structure comparison with known compounds (Quinn *et al.* 2020), some of which were later found in IBD patients by reverse metabolomics (Gentry *et al.* 2024). More interestingly, clinically designed questionnaires integrate dietary factors into the microbiome, creating a nutri-metabolome research model (Xie *et al.* 2023). In summary, advanced multi-omics methods and computerized technology help better understand diet-induced host-microbiota interaction in IBD.

### Diet-induced dysbiosis and IBD

The Western diet is characterized by increased caloric intake from simple carbohydrates and long-chain fatty acids, with less fiber and more food additives (Cordain *et al.* 2005), which is highly associated with IBD from clinical and basic experimental perspectives (Ananthakrishnan *et al.* 2014; Guo *et al.* 2024; Hou *et al.* 2011; Martinez-Medina *et al.* 2014).

Simple carbohydrates are sugars easily absorbed by the small intestine, such as glucose, fructose, and sucrose syrup added to soft drinks (Gouyon *et al.* 2003). Among them, fructose is recognized as the metabolic toxin on the gut-liver axis, which can lead to impaired intestinal barrier function, dysbiosis, and increased entrance of bacterial toxins into the bloodstream (Febbraio and Karin 2021). The exacerbation of enteritis by increased fructose exposure in the Western diet was demonstrated in mice models. For example, high-fructose corn syrup worsens intestinal inflammation through the imbalance in T-cell differentiation mediated by disturbances in microbial bile acids (BAs) (Zhou *et al.* 2023). The same immune dysregulation can also be seen in the high-glucose diet (Wegorzewska *et al.* 2019). In addition, simple carbohydrates also lead to impaired intestinal barriers by increasing mucus-degrading bacteria (Khan *et al.* 2020; Montrose *et al.* 2021) or indirectly via microbial metabolites (Laffin *et al.* 2019; Zhou *et al.* 2023) and metabolic reprogramming (Jones *et al.* 2021), and even consequently increase exposure to lipopolysaccharide (LPS) (Jones *et al.* 2021; Song *et al.* 2023) inducing more severe immune disorders. Recently, we developed a system to mine microbial enzymes and found that bacterial-derived host isoenzymes cause glucose metabolism disorders when the intestinal barrier is defective. Patients with IBD may have increased interactions between these isoenzymes, which are needed to evaluate the microbiota's potential effects (Wang *et al.* 2023).

Western dietary lipids are characterized by increased saturated fatty acids (SCFAs), trans fatty acids (TFAs), and a high ω-6/-3 polyunsaturated fatty acids (PUFAs) ratio (Cordain *et al.* 2005). High levels of saturated fatty acids such as palmitic acid lead to a compromised bowel barrier (Bashllari *et al.* 2023; Gori *et al.* 2020).TFA is likewise recognized as a pro-inflammatory factor in IBD (Higashimura *et al.* 2020; Yao *et al.* 2017). In the same context, ω3-PUFA can produce counteracting (Escoula *et al.* 2019) or opposite (Yao *et al.* 2017) effects with these pro-inflammatory fatty acids. Meanwhile, IBD patients present lower levels of ω3-PUFA (Zhou and Zhou 2024), consistent with its traditional role of inhibiting inflammation (Wall *et al.* 2010). However, two recent studies have suggested the pro-inflammatory potential of ω3-PUFA (Kroschwald *et al.* 2018; Rohwer *et al.* 2021). ω6-PUFA can mediate inflammation in IECs that could be limited by GPX4, whose expression is decreased in the mucosa of CD patients to prevent lipid peroxidation and iron death (Mayr *et al.* 2020). The same protective effect alleviates specific AIEC-mediated damage to IECs, and it is of concern that at this point inflammation is worsened by either ω3- or ω6-PUFA supplementation (Wen *et al.* 2023). The clinical role of the PUFA needs to be further explored.

Moreover, dietary quantity and rhythm may influence the progression of IBD. For instance, calorie-restricted diets increase fecal Lactobacillus and Bifidobacterium in mice (Fan *et al.* 2023; Fraumene *et al.* 2018), which may help protect the gut from injury by producing lactic acid to support the growth of ISCs (Lee *et al.* 2018; Wu *et al.* 2024). Additionally, dietary rhythms can cause microbial fluctuation (Thaiss *et al.* 2014; Zarrinpar *et al.* 2014) or affect the expression of genes about the intestinal biological clock to impact the metabolome (Heddes *et al.* 2022). In conclusion, it's important to consider the dietary composition, consumption, and rhythms of microbial fluctuations to understand better how diet-microbial interactions affect gut health and IBD.

### Nutrient-related microbial interactions

Niches reflect the roles and functions of species in ecosystems, including resource use and interactions with other biotic and abiotic factors (Shepherd *et al.* 2018). In this structure, microbiota uses dietary components to colonize and survive, developing chronic competitive or cooperative relationships for resources, while dynamic homeostatic interactions are closely linked to host health (Wilde *et al.* 2024). The species output positive, negative, or neutral effects that can be combined to form a variety of interactions (Faust and Raes 2012). The recent emergence of IBD-related research focuses on competition and cross-feeding among microbiota (including mutualistic, commensalism, or mutualistic interactions) (Culp and Goodman 2023).

Commensalism primarily refers to a one-way positive feeding relationship. As in phytate metabolism, *Mitsuokella jalaludinii* acts as a phytate degrader and metabolizes the antimicrobial substance 3-hydroxypropionate but can be utilized by *Anaerostipes rhamnosivorans* to achieve commensalism. The end-product propionate activates tight junction genes to maintain the intestinal barrier (De Vos *et al.* 2024). Besides, viral research suggests that phages colonize the mucus layer by infecting bacteria, forming a commensal relationship, and reducing bacterial virulence (Almeida *et al.* 2019). So, it's thoughtful to optimize co-culture techniques for microorganisms with complementary metabolic pathways that can share resources and protect the gut.

Moreover, a report has proposed an integrated framework of four trophic levels, focusing on the mutualistic relationship in polysaccharide-short chain fatty acid (SCFA) metabolism (Culp and Goodman 2023). However, this kind of balance can be disturbed by other species. For example, in a pair of mutualistic relationships, phages attacking their host *Escherichia coli* to release cellular debris or promote the evolution of host resistance to secrete more acetate results in more favorable growth of the non-host *Salmonella enterica* (Fazzino *et al.* 2020), which contributes to IBD etiology (Schultz *et al.* 2017).

Furthermore, relationships between microbes may be subverted by resource abundance. One study found that increasing nutrients may induce competition among partner microbes (Hoek *et al.* 2016), suggesting the potential effect of diet on microbial interactions. However, there is no need to worry about this shift as competition can maintain global homeostasis by inhibiting the overgrowth of the dominant microbiota (Hoek *et al.* 2016). SCFAs can create an acidic gut environment, which may be detrimental to pathogenic bacteria that are not adapted to grow at low pH levels (Fukuda *et al.* 2011).

In summary, the impact of nutrient-induced microbial factors on IBD can be enriched from individual microbiota to analyze microbial interactions.

## DIETARY-DERIVED MICROBIAL METABOLITES

In this section, we aim to analyze the impacts of three dietary-derived microbial metabolites on intestinal homeostasis, which have been the focus of ongoing discussions. We will highlight their effects on intestinal immunity, and the mucosal barrier, and particularly explore additional factors that could inspire new avenues for research on the origins and treatment of IBD.

### Short-chain fatty acids

Humans possess a finite amount of carbohydrate-active enzymes (CAZymes) (Ross *et al.* 2024). Therefore, the breakdown of dietary fiber relies on the fermentation by colonic microbiota (Ross *et al.* 2024), mainly Clostridia and Bacteroidia, producing short-chain fatty acids (SCFAs), including acetate, propionate, and butyrate (Litvak *et al.* 2018; Morrison and Preston 2016). Among them, butyrate is the main fuel for colonocytes and increases oxygen consumption through PPAR-γ-dependent activation of mitochondrial β-oxidation (Kelly *et al.* 2015). The resulting physiological hypoxia sustains obligate anaerobic bacteria to beneficial fermentation, fostering a mutually goal-directed exploitation between the host and microbiota (Litvak *et al.* 2018). What counts is low oxygen levels stabilize hypoxia-inducible transcription factors (HIFs) (Kelly *et al.* 2015; Ma *et al.* 2022). HIF-2α could upregulate MUC2 expression in goblet cells (Ma *et al.* 2022). Furthermore, HIF-1α acts as a prolyl hydroxylase (PHD) inhibitor, preventing cell apoptosis (Tambuwala *et al.* 2010). Studies have shown that a synthesized PHD inhibitor promotes tissue recovery in murine colitis (DeFrates *et al.* 2024), while butyrate directly inhibits PHD (Wang *et al.* 2021), highlighting its potential therapeutic effects on IBD. In brief, HIFs take a warning signal to address inflammation, and its downstream target genes preserve the intestinal epithelium (Colgan and Taylor 2010). In contrast, treatment with streptomycin can reduce butyrate production by Clostridia, leading to colonic anaerobic glycolysis favoring pathogen colonization at higher oxygen levels, such as *Candida albicans* (Jangi *et al.* 2024; Lewis *et al.* 2017; Savage *et al.* 2024; Sokol *et al.* 2017), which increases in IBD patients (Imai *et al.* 2019). *Candida albicans* is more invasive with the unbalanced intestinal barrier (Yan *et al.* 2013), potentially forming a vicious cycle of IBD inflammation and damage aggravation mediated by SCFA deficiency.

This metabolic shift forms the butyrate paradox (Salvi and Cowles 2021). Rather than fuel in undifferentiated cells, such as tumor cells favoring glucose, butyrate shows carcinostasis by controlling excessive proliferation through inhibiting HDAC (histone deacetylases). Whereas for intestinal stem cells (ISCs), although the concentration gradient reaches a low peak at the crypt, butyrate still suppresses ISCs proliferation by downregulating HDAC-mediated FoxO3 (Kaiko *et al.* 2016). More specifically, it has recently been shown that butyrate alleviates DSS-induced colitis by inhibiting HDAC to downregulate hexokinase 2 expression, the rate-limiting enzyme of glycolysis that is highly expressed in IBD (Hinrichsen *et al.* 2021). Hence SCFA level in the crypt niche can reprogram colonocytes and regulate exposed ISCs. This has significance for screening drugs to improve intestinal repair in IBD.

Due to low fiber in the Western diet, the cecum and colon microbiota lack nutrients from microbiota-accessible carbohydrates (MACs), reducing specific microbiota (Makki *et al.* 2018). Reduced fecal SCFAs are often found in IBD patients (Quinn-Bohmann *et al.* 2024; Vernia *et al.* 1988), particularly those with UC, accompanied by decreased butyrate-producing strains like *Roseburia hominis* and *Faecalibacterium prausnitzii* (Machiels *et al.* 2014). Therefore, fiber deprivation leads to a metabolic shift in the microbial community, causing them to consume the host-produced mucin (Desai *et al.* 2016; Neumann *et al.* 2021; Wolter *et al.* 2024).

Conversely, adequate fiber-derived SCFAs can fill in the gap by accelerating continuous mucus production by activating the FFAR2 receptor (Holmberg *et al.* 2024) and promoting goblet cell differentiation (Wang *et al.* 2024b), enhancing epithelial surface fluidity, and especially stimulating the clearance of problematic microorganisms during immune activation. Moreover, SCFAs potentiate intestinal resistance by fostering the antimicrobial peptide (Zhao *et al.* 2018). Furthermore, butyrate and propionate have been reported to intensify tight junctions, thus strengthening the physical defense (Yan and Ajuwon 2017). In addition, SCFAs have immunomodulatory roles by binding to G-protein-coupled receptors (GPCRs) to modify T-cell homeostasis against intestinal inflammation, stimulate the proliferation of colonic T regulatory (Treg) cells via GPR43 (Smith *et al.* 2013), and promote the differentiation of Treg and IL-10-producing T cells by binding to GPR109a (Singh *et al.* 2014). SCFAs also mediate cytokines formation, like upregulating IL-10 and IL-22 through GPRs (Sun *et al.* 2018; Yang *et al.* 2020). Additionally, less-studied pentanoate could induce IL-10 production in lymphocytes and inhibit IL-17A production, inhibiting autoimmunity (Luu *et al.* 2019).

In short, SCFAs may help treat IBD by controlling the balance of microorganisms in the gut. Besides, branched-chain SCFAs, such as isopropionic acid, have anti-inflammatory effects in IBD treatment (Pereira *et al.* 2024). Further research into non-classical SCFAs-producing microbiota may help unlock this mystery.

### Bile acids

Bile acids (BAs) are synthesized de novo in hepatocytes and then conjugated to glycine or taurine to form conjugated bile acids (CBAs). These primary bile acids (PBAs) are released into the small intestine as detergents during meals. Approximately 95% of PBAs are reabsorbed in the ileum through the enterohepatic circulation, while the remainder escapes to the colon and undergo enzyme-mediated microbial biotransformation that finally generates secondary bile acids (SBAs) (Ridlon and Gaskins 2024).

On the other hand, BAs can alter the microbial composition. Ursodeoxycholic acid (UDCA) has demonstrated potential therapeutic effects in IBD (Gao *et al.* 2021). One possible mechanism is upregulating MUC2 expression provides a supportive mucin layer for beneficial bacteria like *Akkermansia muciniphila*, which in turn, promotes resistance to colitis (He *et al.* 2023). Another view worth noting is diet could induce dysbiosis by disturbing the BA profile. A diet high in saturated fatty acids enriched in taurocholic acids contributed to the flourishing of taurine-utilizing *Bilophila wadsworthi*, and worsened murine colitis (Devkota *et al.* 2012). Moreover, during metabolic processes, *Bilophila wadsworthi*, as the sulfate-reducing bacteria (SRB) found in high quantities in the feces of IBD patients, can produce H2S, which harms the intestinal barrier (Sorrentino *et al.* 2020).

In this way, it’s easy to predict that microbiota-mediated enzymatic dysfunction leads to metabolic disorders of BAs. For example, impaired microbial desulphation activities cause the accumulation of proinflammatory 3-OH-sulphated BAs offering a vicious circle for IBD (Duboc *et al.* 2013). Hence, BAs and microbiota mutually modulate to influence gut ecology.

In IBD clinical cohorts, alterations in the BA profile are characterized by elevated PBAs and reduced SBAs in fecal samples (Battat *et al.* 2023; Jagt *et al.* 2022). Recent findings revealed that SBAs are dedicated to the remission of murine colitis, as 12-ketolithocholic acid could inhibit the secretion of IL-17A by type 3 innate lymphoid cells (ILC3) through upregulating Vitamin D receptor (VDR) (Li *et al.* 2023). And surprisingly, deoxycholic acid (DCA) and lithocholic acid (LCA) are reported to stimulate ISC proliferation as Takeda G protein-coupled receptor 5 (TGR5) agonists (Sorrentino *et al.* 2020). However, the cytotoxicity of SBAs and their association with GI tumorigenesis underscore the need for further clinical investigation into their therapeutic potential for IBD (Ridlon and Gaskins 2024). Moreover, several LCA derivatives are found accumulating in centenarians, with the newly identified isoalloLCA effectively inhibiting pathogenic *Clostridium* *difficile* (Sato *et al.* 2021). Of particular concern is their role in T-cell regulation. For instance, 3-oxoLCA directly binds to RORγt, thereby inhibiting Th17 cell differentiation or being converted into isoalloLCA by *Bacteroides* species with increasing differentiation of Foxp3+ Tregs cells through NR4A1 (Hang *et al.* 2019). Besides, IBD patients have the excessive fecal accumulation of CBAs (Duboc *et al.* 2013), which in T effector (Teff) cells induces oxidative stress and leads to CD-like ileitis in *Rag1*^-/-^ mice, while upregulation of xenobiotic transporter Mdr1 expels excess (Cao *et al.* 2020). However, a high-fat diet impairs cellular BA detoxification (Zheng *et al.* 2024b), suggesting dietary undermining effects on the host’s physiological control of BA levels could aggravate colitis.

In parallel to the classical CBAs, novel amido BAs, known as microbially conjugated bile acids (MCBAs), have been discovered (Quinn *et al.* 2020). MCBA generation has recently been shown by bile salt hydrolases (BSH) (Guzior *et al.* 2024; Quinn *et al.* 2020; Rimal *et al.* 2024). However, classically known for deconjugation, hypofunction of BSH is attributed to increased CBAs in IBD (Duboc *et al.* 2013). It therefore enlightens the complexity of function and substrate selectivity in different states of bacterial-derived enzymes. Notably, IBD-associated MCBAs and their microbial producers were effectively identified using reverse metabolomics (Gentry *et al.* 2024). While extensive research already exists on how bile acids bind to receptors to influence host immunity and metabolism (Fleishman and Kumar 2024), the discovery of novel bile acids highlights the need for further investigation into the microbiota, as well as enzyme-related genes and catalytic mechanisms, which provide a theoretical foundation for precision therapies targeting IBD.

Remarkably, our team has recently established a platform for exploring microbiota and microbial enzymes, leading to the discovery of uncharacteristic 3-succinylated cholic acid (3-sucCA), produced by β-lactamase from *Bacteroides uniformis* (Nie *et al.* 2024). This novel BA promotes the growth of *Akkmansia muciniphila*, indicating potential mucosal protective effects akin to UDCA (He *et al.* 2023). Since diet and microbiota can both influence the BA profile, further research into diet-mediated BA-microbiota interactions is warranted.

### Tryptophan and indole derivatives

There are three pathways in tryptophan metabolism, which regulate intestinal homeostasis under the joint action of host and microbiota (Agus *et al.* 2018). For IBD patients, the indoleamine 2,3-dioxygenase (IDO) pathway is enhanced, while the aryl hydrocarbon receptor (AhR) pathway is down-regulated (Agus *et al.* 2018), which can also be found in high-fat diet mice (Laurans *et al.* 2018). Notably, knockout or inhibition of the IDO pathway improves intestinal injury and its metabolic disorder in mice (Laurans *et al.* 2018), while *Ido1*^-/-^ mice develop aggravated colitis (Takamatsu *et al.* 2013). This contradiction leads to controversy over the efficacy of IDO inhibitors. In addition, disruption of the circadian oscillation of tryptophan metabolism mediated by microbiota can lead to impaired barrier function (Gheorghe *et al.* 2024). The relationship between diet-induced dysbiosis of microbiota and IBD needs further investigation.

Indole and its derivatives have been widely demonstrated to inhibit intestinal inflammation in mice through the AhR-IL22 mechanism (Geng *et al.* 2018; Monteleone *et al.* 2011; Renga *et al.* 2022; Zelante *et al.* 2013), probably owing to the tissue repair and antimicrobial properties of IL-22 (Witkowski *et al.* 2018). In addition, indoleacetic acid (IAA) was recently found to enhance intestinal barrier function by enhancing mucin sulfation (Witkowski *et al.* 2018). Other indole derivatives can also reduce intercellular space or permeability (Scott *et al.* 2020). These are all mediated by AhR receptors exhibiting functional diversity. Oral supplementation of Lactobacillus reuteri and tryptophan is proved to alleviate colitis through the AhR-IL22 pathway, but not in *AhR*^-/-^ mice (Islam *et al.* 2017; Lamas *et al.* 2016). The reduced AhR gene expression in IBD (Monteleone *et al.* 2011) may limit the efficacy of dietary tryptophan supplementation.

In particular, dietary intervention could also alter the balance of tryptophan metabolism by mediating microbiota competition, indicating the substrate-dependent property of microbiota metabolic output. For example, providing *E. coli* with fiber-derived monosaccharides can reduce its competitiveness in tryptophan metabolism and harmful indole production, thereby increasing the production of indole propionic acid (IPA) and indole lactic acid (ILA) by other bacteria that protect against intestinal inflammatory damage (Lamas *et al.* 2016). There is also a crossfeeding pattern of tryptophan metabolism among microbiota, with Lactobacillus metabolizing tryptophan to ILA, up-regulating the expression of key bacterial enzymes and the abundance of Clostridium, thereby enhancing IPA and IAA production (Wang *et al.* 2024a). These indicate that dietary interventions can combine multiple nutrients or probiotic-mediated amplification effects to increase efficacy.

### High-molecular-weight bacterial compounds

Recently, the high-molecular-weight compounds from gut microbes have been found to play key roles in regulating gut barrier functions. For instance, LPS (lipopolysaccharide) strongly induces pro-inflammatory responses through the host myeloid-differentiation-2/Toll-like receptor 4 (MD-2/TLR4) receptor complex. However, a weak agonist LPS derived from *Bacteroides vulgatus* mpk (BVMPK) reduces inflammation in IBD mice by inducing endotoxin tolerance and semi-maturation in CD11c+ cells (Steimle *et al.* 2019). Besides, lipoteichoic acid (LTA), another cell wall component of *Lactobacillus paracasei* D3-5 strain (D3-5), enhances mucin expression and ameliorates age-related leaky gut and inflammation (Wang *et al.* 2020b). Another research found LTA from *Lactobacillus rhamnosus* GG promotes mesenchymal stem cell migration to the crypt for injury repairment by binding to TLR2 of macrophages (Riehl *et al.* 2019). Except for lipid-related molecules, peptidoglycan (PGN), polysaccharide purified from *Lactobacillus salivarius* Ls33, rescued mice from colitis in an IL-10-dependent manner (Macho Fernandez *et al.* 2011). And as for protein, the outer membrane protein Amuc_1100 from *Akkermansia muciniphila* is reported to improve colitis, together with tumourigenesis by expanding CTLs in the colon (Wang *et al.* 2020a). Therefore, when exploring the effects of microbial metabolites on IBD, the molecules of microbial origin should also be taken into account.

## EXCLUSIVE ENTERAL NUTRITION

Exclusive enteral nutrition (EEN) is an intensive dietary regimen of special liquid replacements given orally or by tube, excluding other foods, to supply all the caloric and intact nutrient elements the patient requires. Several guidelines have recommended EEN as a first-line therapy for pediatric patients with mild-to-moderate active CD and generally requires six to eight weeks to induce clinical remission. For adults and patients with UC, traditional drug therapy is still preferred over EEN (Bischoff *et al.* 2023; Hashash *et al.* 2024; Lamb *et al.* 2019). EEN showed a clinical remission rate of about 60%–80% compared with conventional corticosteroid therapy but has demonstrated a significantly superior submucosal healing rate in numerous randomized controlled trials (Hashash *et al.* 2024; Runde *et al.* 2023; Yu *et al.* 2019), which is considered to be a new therapeutic indicator in the future. Besides, immunosuppressive agents and biologics, in combination with EEN interventions, have been shown to increase the rate of clinical remission (Zhou *et al.* 2024) and mucosal healing (Hojsak *et al.* 2020) and reduce postoperative recurrence (Duan *et al.* 2024). Notably, the inclusion of CD patients of all ages in these latest studies, suggests that EEN may have therapeutic potential, even if it is not recommended as a first-line therapy for adults.

From the previous discussion, it can be concluded that diet-derived microbial metabolites have a profound effect on intestinal homeostasis. Therefore, EEN, as a dietary intervention therapy, could theoretically be formulated to play a therapeutic role by modulating dysbiosis. There are many experiments showing that EEN can increase probiotics, decrease pathobionts, or modulate microbial metabolites to protect the intestinal tract (Table 2).

**Table 2:** Microbial changes after EEN intervention

Reference	Model/Cohorts	Microbial composition	Metabolic shifts	Effects
Geesala *et al.* 2024a	TNBS-induced colitis in rats	Alpha diversity↑; *Lactobacillus*↑; *Dubosiella*↑; *Bacteroides*↓; *Enterorhabdus*↓	–	EEN significantly attenuated the increase in IL-17A and interferon-γ in TNBS-colitis rats
Li *et al.* 2022	TNBS-induced colitis in mice	General diversity↓; beneficial microbiota↑; dentrimental microbiota↓; IgA- IgG- coated bacteria↓	–	EEN alleviates intestinal mucosal inflammation (soluble IgA and IgG↓) in TNBS-colitis mice
Jang *et al.* 2021	DSS-induced colitis in mice	*Akkermansia muciniphila*, *Clostridium cocleatum*, *Flintibacter butyricus*, and P*arabacteroides goldsteinii* ↑	Butyrate↑	EEN promote mucin recycling in DSS-colitis mice
Kuffa *et al.* 2023	Tac-DKO (Nod2 + Cybb) mice （Th-1 like CD colitis）	*Mucispirillum schaedleri*↓	Ammonia (DNRA) metabolic pathway↓	The fiber-free diet reduces nutrient availability for mucin-degrading microbes and impairs the metabolic pathway of *Mucispirillum*, leading to its exclusion from the mucus layer and disease remission
Lunken *et al.* 2021	TCR-β deficient mice	*Bifidobacterium* spp.↑; *Anaerostipes caccae*↑; [*Clostridium*] *innocuum* group spp.↓; *Escherichia Shigella* spp.↓	Butyrate↑	EEN with enriched inulin-type fructan treatment had less deterioration of the colonic mucus layer and increased levels of FOXP3^+^IL-10^+^ and ROR$ \mathrm{\gamma }\mathrm{t} $^+^IL-22^+^ CD4^+^ T cells and decreased levels of Tbet^+^IFNγ^+^ and Tbet^+^TNF^+^ CD4^+^ T cells
Zeng *et al.* 2024	DSS-induced colitis and *Il10*^-/-^spontaneous colitis mouse models	*Faecalibaculum rodentium*↑	Histidine↑	EN-induced *Faecalibaculum rodentium* accumulation protects against colitis in mice via gut bacteria-mediated histidine biosynthesis
Lv *et al.* 2023	27 newly diagnosed pediatric patients with CD and 27 healthy controls	Firmicutes bacteria↑	Secondary bile acid↑	The intestinal microbial structure and secondary bile acid metabolism were significantly impaired in the patients, but returned to normal levels after two months of EEN treatment
Pereira *et al.* 2024	SM14 (14 species of sequenced and metabolically characterized human gut bacteria) into GF *Il10*^-/-^ mice	*Eubacterium rectale*↑	Isobutyrate↑; SCFAs↓	Fiber-free enteral nutrition diet inhibits inflammation by increasing the anti-inflammatory metabolite isobutyric acid despite causing mucus erosion
Metwaly *et al.* 2023	CD-like ileal inflammation in two mouse models	segmented filamentous bacteria (SFB)↓	–	EEN-like purified diet antagonized SFB colonization and prevented disease development in TNFΔARE mice
Xiao *et al.* 2022	DSS-induced colitis in mice; twelve enrolled pediatric CD	*Clostridium innocuum*↑; *Hungatella hathewayi*↑	Hyocholic acid (HCA)↑	EEN improved BA dysmetabolism, with some enriched BAs, including HCA, strongly associated with decreased severity of both in CD mice and cohort
TNBS: trinitro-benzene-sulfonic acid; DSS: dextran sulfate sodium; GF mice: germ-free mice				

In clinical application, pediatric patients could be divided into responders and nonresponders according to efficacy with differences in microbiome and metabolome before treatment (Diederen *et al.* 2020; Nichols *et al.* 2024), or whether showing specific fluctuation (Runde *et al.* 2023). It is suggested that the targeted microbiota therapy of EEN needs a certain premise that diversity and dynamics brought by the microbiota community and individuation are of great importance to developing individualized EEN formulas. However, there are also clinical studies showing that EEN reduces microbial diversity (Beauchamp-Walters *et al.* 2023; Diederen *et al.* 2020), indicating the limitations in interpreting the effective mechanism of EEN with only a single perspective.

In addition, a study of 61 EEN formulas found some contained “harmful ingredients”, like food additives which did not worsen CD or impact remission rates during therapy (Logan *et al.* 2020). Meanwhile, the classic EEN formula lacks fiber (Logan *et al.* 2020). This leads to a situation whereby the time EEN reaches the inflamed colon in patients with UC, most of the beneficial nutrients have already been absorbed in the small intestine, possibly partially explaining the poor outcome in UC (Lunken *et al.* 2021). In previous discussions, the Western diet results in gut damage due to fiber deficiency, which contradicts the efficacy of EEN. Interestingly, two recent studies partially explain this phenomenon. The first reported that a low-fiber diet (represents EEN) decreased the levels of SCFAs but increased the levels of branched-chain fatty acids (BCFAs) in *Il10*^-/-^ mice (Pereira *et al.* 2024), in which this metabolic shift was also observed from a previous clinical cohort (Jatkowska *et al.* 2023). In this study, it may be explained that when fiber-derived SCFAs are reduced, other metabolites play a beneficial role. The generation of isobutyrate counteracts the lethal colitis driven by the Th1 immune response (Pereira *et al.* 2024). Another study related to microbial crossfeeding. The interspecies H_2_ transfer in the metabolic framework of fibers and SCFAs is a typical, H_2_ producer providing electron donor for H_2_ consumers, which has profound effects on colonic mucosal homeostasis and inflammation (Litvak *et al.* 2018). This framework is ideal for understanding the mechanisms of reduced fiber supply-mediated mucosal protection. The disruption of the H_2_ economy between mucus-degrading bacteria and the IBD-associated mucus-inhabiting bacterium *Mucispirillum* affects the latter's metabolism and colonization (Fitzgerald and Sorbara 2023). Moreover, there are likewise many anaerobic bacteria such as SRB that demonstrate hydrogenotrophic respiration, which enriched in IBD produce H_2_S to impair intestinal homeostasis (Figliuolo *et al.* 2018).

In this way, it is not sufficient to analyze the effective mechanism of EEN from its nutrient composition alone. Microbial oscillations due to exposure to diurnally fluctuating metabolites and effects on intestinal physiology (Thaiss *et al.* 2016; Thaiss *et al.* 2014). As with diurnal fluctuations in segmented filamentous bacteria (SFB) adhesion leads to oscillating levels of intestinal antimicrobial peptides, resulting in changes in intestinal defenses throughout the day (Geesala *et al.* 2024b). In contrast, the inflammatory effect of SFB colonization on the mouse intestine can be inhibited by EEN-like purified diets (Li *et al.* 2022). Whether EEN efficacy is influenced by interfering with microbial rhythms needs to be explored in larger clinical and basic experiments. Furthermore, clinical cohort studies have found no significant differences in the efficacy of different EEN delivery modes and formulation characteristics in children with CD (Hojsak *et al.* 2020; Rubio *et al.* 2011). However, a continuous supply of nutrient solution over 24 hours may be more conducive to sustained repair of the intestinal mucosa in a stable environment (Rubio *et al.* 2011). Instead, split oral intake is more consistent with normal dietary patterns and so has the opportunity to improve patient palatability and tolerance, which is often considered a key factor in adult outcomes (Lamb *et al.* 2019).

**Figure 1:**
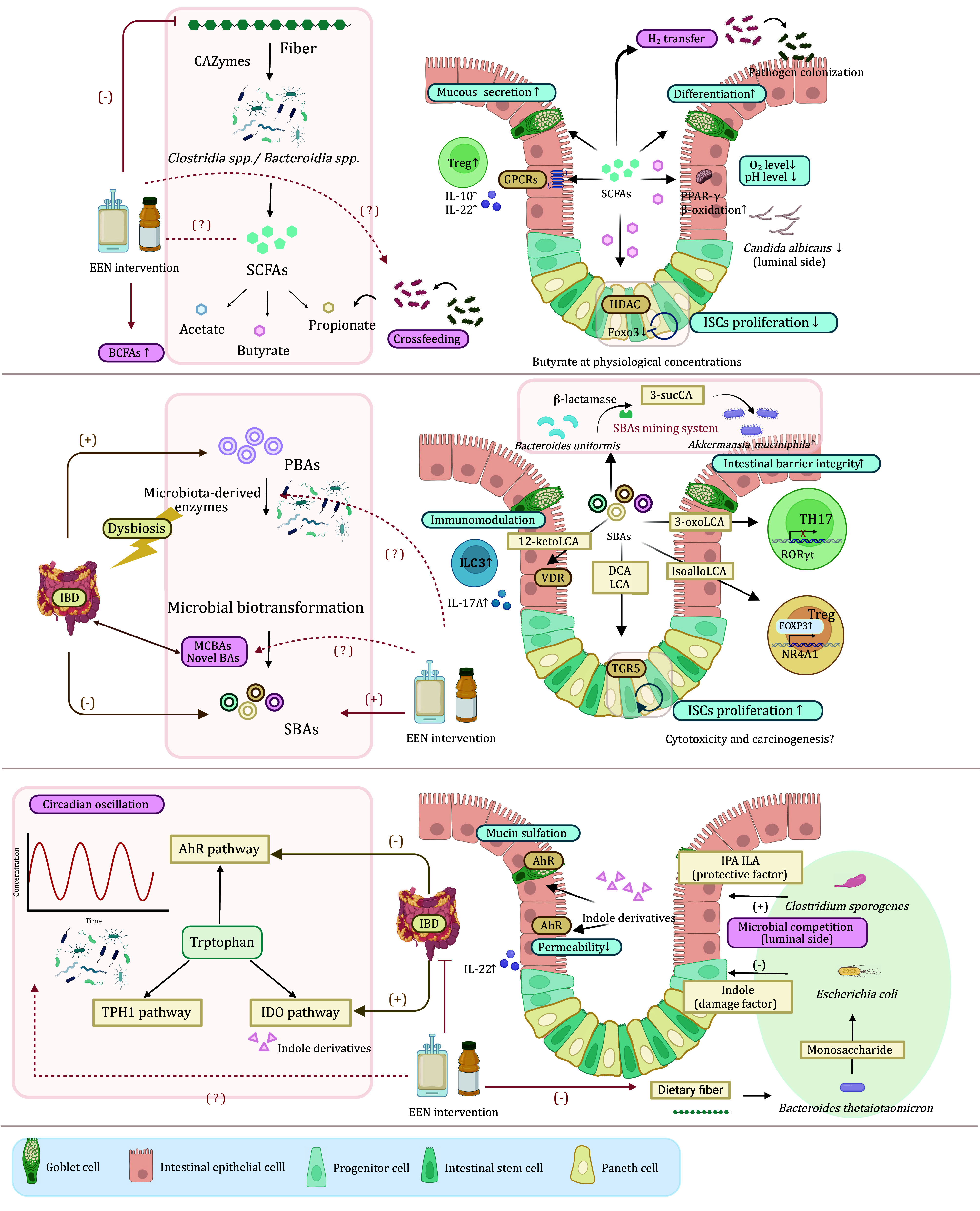
Nutrient-derived microbial metabolites influence intestinal homeostasis. Dietary components are utilized by the intestinal flora and generate multiple metabolites such as SCFAs, BAs, and indole derivatives. These metabolites are capable of influencing intestinal physiology, especially on the integrity of the intestinal barrier by facilitating mucus production in goblet cells, modulating immune cell composition and cytokine production, or affecting stem cell niche. In addition, nutrients can also induce microbial social behaviors such as crossfeeding and competition. The diet-microbiota mechanisms involved in the EEN intervention have been labeled in the figure, where a plus sign implies an increase, a minus sign is thought to be a decrease, and a question mark indicates an urgently needed research direction

In summary, the underlying mechanisms of EEN require a broader perspective to break through existing contradictions with traditional perceptions, which helps to optimize the formulation or delivery method for wider and more efficient applications (Fig. 1).

## SUMMARY AND PERSPECTIVES

Dysbiosis in patients with IBD has been widely demonstrated. Although the disease-characterized microbiome is not yet uniformly known, a study has shown the clinical promise of using combinations of multiple bacterial markers as a noninvasive diagnostic method for IBD (Zheng *et al.* 2024a). Further research is necessary to map the specific microbiome of IBD for targeted therapy. Besides, by reviewing the effects of multiple diet-microbial interactions on gut physiology, the principles of dietary intervention therapies for IBD can be better analyzed, as well as provide a theoretical basis for microbe-targeted precision medicine. However, the mechanism of EEN is still not fully elucidated, especially in its formulation-induced diet-microbial interactions, which leaves behind puzzles that hinder the possibility of optimizing the formula to improve clinical efficacy. While most studies have focused on observing how dietary changes affect gut immunity and mucosal barriers through microbial changes, existing reviews have indicated that the dietary metabolome can also impact ISCs (Shay and Yilmaz 2024). Therefore, more research is needed in the future to construct a diet-microbiota-ISC axis to delineate the boundary between barrier repair and tumorigenesis. In addition, inter-species communication during diet-induced microbial metabolism also has an important impact on shaping individual differences in the microbiome (Culp *et al.* 2024), and this community behavior has been shown to influence efficacy in drug metabolism (Garcia-Santamarina *et al.* 2024). This requires researchers to extend the observation of individual colony changes to the construction of microbial interaction networks when exploring the mechanisms of dietary intervention therapies.

## Conflict of interest

Xinran Wang, Yiran Wang and Lulu Sun declare that they have no conflict of interest.
